# Carbon monoxide increases utero-placental angiogenesis without impacting pregnancy specific adaptations in mice

**DOI:** 10.1186/s12958-020-00594-z

**Published:** 2020-05-14

**Authors:** Megan A. Dickson, Nichole Peterson, Karalyn E. McRae, Jessica Pudwell, Chandrakant Tayade, Graeme N. Smith

**Affiliations:** 1 Department of Biomedical and Molecular Sciences, Queen’s University, Kingston, ON K7L 3N6 Canada; 2 Department of Obstetrics and Gynaecology, Queen’s University, Kingston Health Sciences Centre, 76 Stuart St, Kingston, K7L 2V7 Canada

**Keywords:** Carbon monoxide, Murine pregnancy, Implantation site, Angiogenesis, Preeclampsia

## Abstract

**Background:**

Cigarette smokers have a reduced risk of developing preeclampsia, possibly attributed to an increase in carbon monoxide (CO) levels. Carbon monoxide is a gasotransmitter that has been implicated in maintaining vascular tone, increasing angiogenesis, and reducing inflammation and apoptosis at physiological concentrations. Moderately increasing CO concentrations may have therapeutic potential to prevent or treat preeclampsia; however, the effects of CO on pregnancy are under studied. Our objective was to investigate the effect of CO on major angiogenic and inflammatory markers in pregnancy, and to evaluate the effect of CO on indicators of placental health.

**Findings:**

Pregnant CD-1 mice were constantly exposed to either ambient air or 250 ppm CO from conception until gestation day (GD)10.5 or GD16.5. Using a qRT-PCR array, we identified that CO increased expression of major angiogenic genes at the implantation site on GD10.5, but not GD16.5. Pro-inflammatory cytokines in the plasma and tissue lysates from implantation sites in treated mice were not significantly different compared to controls. Additionally, CO did not alter the implantation site phenotype, in terms of proliferative capacity, invasiveness of trophoblasts, or abundance of uterine natural killer cells.

**Conclusions:**

This study suggests that CO exposure is pro-angiogenic at the maternal-fetal interface, and is not associated with demonstrable concerns during murine pregnancy. Future studies are required to validate safety and efficacy of CO as a potential therapeutic for vascular insufficiency diseases such as preeclampsia and intrauterine growth restriction.

## Introduction

Preeclampsia (PE) complicates 3–5% of pregnancies and is a leading cause of maternal and fetal morbidity worldwide [1]. Inadequate remodelling of the uterine spiral arteries has been identified in pregnancies complicated by PE [2]. Additionally, women afflicted by PE exhibit increased levels of anti-angiogenic factors both locally in the placenta and systemically [3, 4]. This aberrant vascular phenotype is thought to account for the poor placental perfusion associated with PE.

Smoking cigarettes has been shown to reduce the risk of developing PE by as much as 33% [5]. This risk reduction is not observed in smokeless tobacco users (snuff), suggesting that a combustible byproduct of cigarette smoke is responsible for this effect [6]. We hypothesize that carbon monoxide (CO), the major combustible product of cigarette smoke, is the molecule that confers this reduced risk. Increasing CO, either by endogenous or exogenous means, may offer prophylaxis for the prevention or possible treatment for PE.

The potential of CO as a therapeutic is related to its vasodilatory [7], angiogenic [8], anti-apoptotic, and anti-inflammatory [9] properties, with the aim that it could improve utero-placental perfusion and function. We have previously demonstrated that CO exposure decreases syncytiotrophoblast apoptosis and secondary necrosis in an in vitro placental model of hypoxia-reoxygenation insult [10]. Additionally, CO exposure increases utero-placental vascular branching and diameter in mice [8]. Furthermore, in the adenovirus soluble Fms-like tyrosine kinase-1 (AdsFlt-1) mouse model of PE, CO treatment normalized blood pressure and renal function [11]. Collectively, this evidence suggests that CO exposure may benefit women with PE who exhibit vascular compromise.

Further research is needed to demonstrate the effect of CO on normal pregnancy specific adaptations. We have previously reported that chronic maternal CO delivery at 250 ppm does not alter placental or fetal weight, or result in placental histomorphological changes during murine pregnancy [12]. Using the same dose and method of CO delivery, we hypothesize that CO will increase angiogenesis through major angiogenic pathways without any demonstrable adverse outcomes in a healthy murine pregnancy. The specific aims of this study were to a) determine if CO altered angiogenic pathways at the implantation sites, b) determine if there is an effect of CO on the local and systemic cytokine profiles of pregnancy, and c) determine the effect of CO on markers of placental function, including cellular proliferation, trophoblast invasion, and uterine natural killer (uNK) cell infiltration.

## Methods

### Animals

All experimental procedures were approved by the Queen’s University Animal Care Committee (Smith, 2016–1635), and conducted in accordance with the Canadian Council on Animal Care guidelines. Female CD-1 mice (Charles River Laboratories, USA) at 5 to 7 weeks were mated with CD-1 males. Dams were placed in a CO-dosing chamber as previously described [12] on gestation day (GD)0.5 and exposed to gaseous 250 ppm CO until euthanasia at GD10.5 (*n* = 5) or 16.5 (*n* = 4). Control mice received ambient air in the chamber until euthanized on GD10.5 (*n* = 5) or 16.5 (*n* = 5). At end-points, implantation sites were randomly selected from uterine horns; once collected, implantation sites were labelled using a system that did not reveal treatment group to blind analysis. Pregnancy outcomes including maternal gestational weight gain, number of implantation sites, number of live fetuses, and the number of resorptions per litter were measured at GD10.5 and GD16.5. All n values reported refer to the number of dams.

### Blood collection

Maternal blood was collected via the submandibular vein on GD0.5 (baseline prior to treatment), GD5.5, GD10.5, and GD16.5 into microcentrifuge tubes containing ethylenediaminetetraacetic acid (EDTA) (Bioshop, Canada). Whole blood was used for hemoglobin (Hb) and carboxyhemoglobin (COHb) measurements. Plasma was isolated via centrifugation (6000 rpm, 6 min) for cytokine analysis.

### Maternal carboxyhemoglobin measurement

Hemoglobin and COHb were measured in whole blood as previously described [12]. Briefly, Hb was measured in duplicate immediately following blood collection using a Hemocue Hb 201 (HemoCue, Sweden). For CO measurements, amber vials (2 mL) (27238 Sigma Aldrich, USA) capped with 8 mm silica septa (C13302 Chromatographic Specialties, Canada), containing 20 μL of 2% 5-sulfosalicyclic acid (S3147 Sigma Aldrich, USA) were prepared. Blood (0.2–1.0 μL) was added to the vials using a gas tight Hamilton syringe and repeater system (Hamilton, USA). Vials were prepared in triplicate, with a “blank” vial containing only 2% 5-sulfosalicyclic acid. Following an incubation period of 45 min under ice, CO concentration was analyzed using a head-space gas chromatograph CO analyzer (Peak Laboratories, USA). Percent COHb was calculated using the measured Hb, measured CO in whole blood, and the CO binding capacity of Hb [13].

### Plasma cytokine analysis

Plasma was diluted 1:2 in phosphate buffered saline (PBS) and analyzed using a commercially available mouse 31-plex cytokine assay (Eve Technologies, Canada) as described previously [14]. The panel included selected cytokines and chemokines: Eotaxin, G-CSF, GM-CSF, IFN*γ*, IL-1α, IL-1ß, IL-2, IL-3, IL-4, IL-5, IL-6, IL-7, IL-9, IL-10, IL-12 (p40), IL-12 (p70), IL-13, IL-15, IL-17A, IP-10, KC, LIF, LIX, MCP-1, M-CSF, MIG, MIP-1α, MIP-1ß, MIP-2, RANTES, TNFα, and VEGF.

### Implantation site protein extraction for cytokine analysis

Two to three implantation sites per dam (*n* = 5 control, *n* = 5 CO) were randomly selected for protein extraction. Protein was isolated with an extraction buffer containing Tris Buffer (20 mM Tris HCl pH 7.5, 0.5% Tween 20, 150 mM NaCl), cOmplete protease inhibitor cocktail (25178600 Sigma Aldrich, USA), and sodium orthovanadate (S6508 Sigma Aldrich, USA). Briefly, each implantation site was homogenized on ice with extraction buffer using a Kontes Pellet Pestle (K749514–0000 Fisher Scientific, Canada) for 30 s. Homogenates were centrifuged at 4 °C (10,000 x g, 10 min) and the supernatants were collected. Protein concentrations for all samples were measured using a DC protein assay (5000116 Bio-Rad Laboratories, USA) and normalized to 1000 μg/mL. The same commercially available assay (Eve Technologies, Canada) described for plasma was used to measure cytokine levels in two to three GD10.5 implantation sites per dam (*n* = 5 control, *n* = 5 CO).

### Implantation site RNA extraction

Total RNA was extracted from one randomly selected implantation site per dam using RNeasy Mini Kit (74104, Qiagen, USA). Briefly, 30 mg of frozen implantation site was disrupted by homogenization and the tissue lysate was centrifuged (15,000 x g, 3 min). The cleared lysate was combined with 900 μL of 70% ethanol, added to a RNeasy mini spin column, and centrifuged (8000 x g, 15 s). The column was washed 3 times with buffer, with centrifugation (8000 x g, 15 s) between each wash. The column was dried by centrifugation (8000 x g, 2 min), and the RNA was eluted with 50 μL of nuclease-free water. RNA concentration and purity was determined using a NanoDrop 2000 (Fisher Scientific, Canada). RNA with a 260:280 absorbance ratio between 1.8 and 2.1 was used for cDNA synthesis. Total RNA was reverse transcribed into cDNA using RT^2^ First Strand Kit (330404 Qiagen, USA).

### Quantitative real time PCR (qRT-PCR)

Custom RT^2^ Profiler Arrays (CLAM28183 Qiagen, USA) were used to quantify mRNA expression of select genes [see Additional Table 1] involved in angiogenic and inflammatory pathways at GD10.5 (*n* = 5 control, *n* = 5 CO) and GD16.5 (*n* = 5 control, *n* = 4 CO) implantation sites. Briefly, 1350 μL of 2x RT2 SYBR Green Mastermix (330503 Qiagen, USA), 102 μL cDNA, and 1248 μL RNase-free water were combined, and 25 μL of the mixture was added to each well of the custom array containing specific primers. PCR was performed on a LightCycler 480 Real-Time PCR System (Roche Applied Science, Canada) using the cycling conditions described by Qiagen (Heat activation at 95 °C for 10 min, followed by 45 cycles of denaturation at 95 °C for 15 s and extension at 60 °C for 1 min with a ramp rate of 1.5 °C/s). SYBR green fluorescent data were collected during each extension cycle, and a melt curve analysis was performed at the end of the cycling program for each array to verify PCR specificity.

### Immunohistochemistry and image analysis

Three implantation sites per dam (*n* = 3 control, *n* = 3 CO) were randomly selected for histological analysis. Immunohistochemistry for Ki67 (1:1000 ab16667 Abcam, USA) and pan-cytokeratin (1:100 ab9377 Abcam, USA) was performed using the BenchMark XT Automated Stainer (Ventana Medical System Inc., USA) as described previously [15]. Staining for *Dolichos biflorus* (DBA) lectin was performed manually [16]. Slides were scanned at 20X using Aperio ImageScope (Aperio, USA). Positive pixel algorithms (ImageScope v10, USA) for blinded analysis of Ki67, cytokeratin, and NIH ImageJ to quantify uNK cells were used [17]. Briefly, to analyze Ki67 staining, an algorithm that detects positive cells stained with 3,3′-Diaminobenzidine (DAB) was used to quantify the number of positive Ki67 cells in both fetal and maternal areas of the implantation site; this was divided by the total number of cells to obtain the percentage of positive Ki67 cells in these regions. Fetal areas were defined as the labyrinth (Lab) and junctional zone (JZ), and maternal areas were defined as the decidua basalis (DB) and mesometrial lymphoid aggregate of pregnancy (MLAp). For cytokeratin staining analysis, a positive pixel algorithm that detects areas stained with DAB was used to quantify the number of pixels comprising stained regions in the DB; this was divided by the total number of pixels in the DB, to obtain the percentage of positivity stained area in the DB. For DBA lectin staining, NIH ImageJ was used to manually count positive cells in three areas (each 0.16mm^2^ at 20X on Aperio ImageScope) in the MLAp and DB of each implant site [2].

### Statistical analysis

Data were analyzed by non-parametric Mann-Whitney U tests, with Bonferroni corrections using GraphPad 6.0 (GraphPad, USA). Fold change of qRT-PCR data was calculated using the ΔΔCt method as described [18], with normalization to reference genes Gusb (GD10.5) and Hsp90ab1 (GD16.5); statistical analysis used Student’s t-tests. *P*-values< 0.05 were considered significant.

## Results and discussion

To confirm CO delivery, maternal %COHb was determined for both exposed and unexposed dams, respectively (14.15 ± 0.57 vs. 0.67 ± 0.01% on GD5.5, 16.58 ± 0.68 vs. 0.65 ± 0.04% on GD10.5, and 17.19 ± 0.97 vs. 0.72 ± 0.04% on GD16.5), consistent with studies by Venditti et al. [8, 12]. These COHb levels are consistent with women who smoke cigarettes during pregnancy having reported %COHb levels of up to 14% [19].

Previous studies have shown that 250 ppm CO did not affect maternal weight gain, or result in fetal demise or alterations in fetal weight [90]. In the present study, we further corroborate these results. There was no difference in maternal weight gain compared to controls from GD0.5 to GD10.5 (control 6.5 ± 1.0 g; CO 6.2 ± 0.5 g) and from GD0.5 to GD16.5 (control 20.6 ± 1.4 g; CO 27.2 ± 2.0 g). No differences were observed in pregnancy outcomes, including the number of implantation sites, live fetuses, and fetal resorptions [see Additional Table 2].

**Fig. 1:**
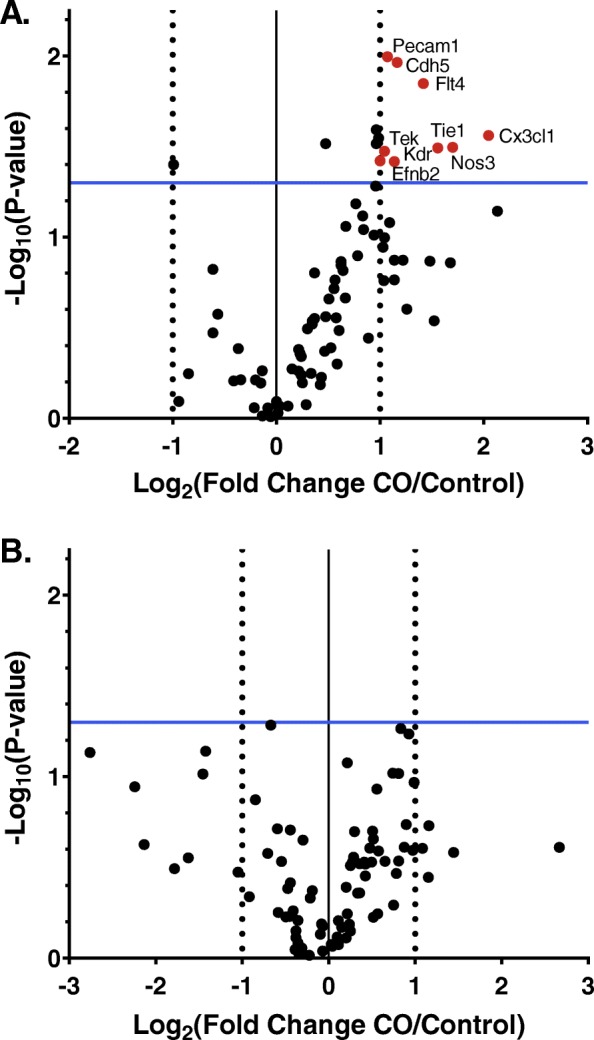
Changes in angiogenic gene expression in (**a**) GD10.5 and (**b**) GD16.5 implantation sites following CO exposure. (**a**) *Kdr, Flt4, Tie2, Tek, Nos, Pecam1, Cdh5, Cx3cl1,* and *Efnb2* expression in GD10.5 implantation sites were upregulated in CO treated mice compared to controls (*n* = 5 control, *n* = 5 CO). (**b**) On GD16.5 there were no significant changes in gene expression (*n* = 5 control, *n* = 4 CO). A 2-fold change and a *p* value of 0.05 were used as cut off thresholds for significant changes in gene expression. Red represents elevated levels of gene expression and black no change. CO, carbon monoxide; GD, gestation day

We measured the expression of select angiogenic related genes at the implantation site using a custom array. Genes involved in angiogenesis, specifically vascular endothelial growth factor receptors (*Kdr* and *Flt4*), angiopoietin receptors (*Tie1* and *Tek*), endothelial nitric oxide synthase (*Nos3*), endothelial cell markers (*Pecam1* and *Cdh5*), fractalkine (*Cx3cl1*), and ephrin-B2 (*Efnb2*) were upregulated at the maternal-fetal interface following CO exposure on GD10.5 (Fig. 1a). No significant changes in gene expression were observed on GD16.5 (Fig. 1b). The VEGF and angiopoietin signalling pathways are essential for placentation [20], and may be targets of CO demonstrated by the increased expression of *Kdr*, *Flt4*, *Tie1*, and *Tek*. These results provide evidence that in murine pregnancy, CO is likely pro-angiogenic and potentiates utero-placental vascular growth midgestation; findings consistent with previous work demonstrating increased branching of the murine utero-placental vasculature following CO exposure [8]. This work demonstrating the effect of CO on angiogenic markers at GD10.5 provides further support that CO, translated into an animal model of PE, may potentially improve the placental angiogenic imbalance, and normalize placental function.

Cytokines are important regulators of embryo implantation and successful pregnancy specific adaptations [21]. However, CO did not result in any measurable changes in the maternal plasma cytokine profiles on GD5.5, GD10.5, or GD16.5 [see Additional Fig. 1]. Additionally, there were no changes in the local cytokine profiles in the implantation sites [see Additional Fig. 2]. Our results suggest that CO does not disrupt cytokine regulated processes in normal pregnancy.

**Fig. 2:**
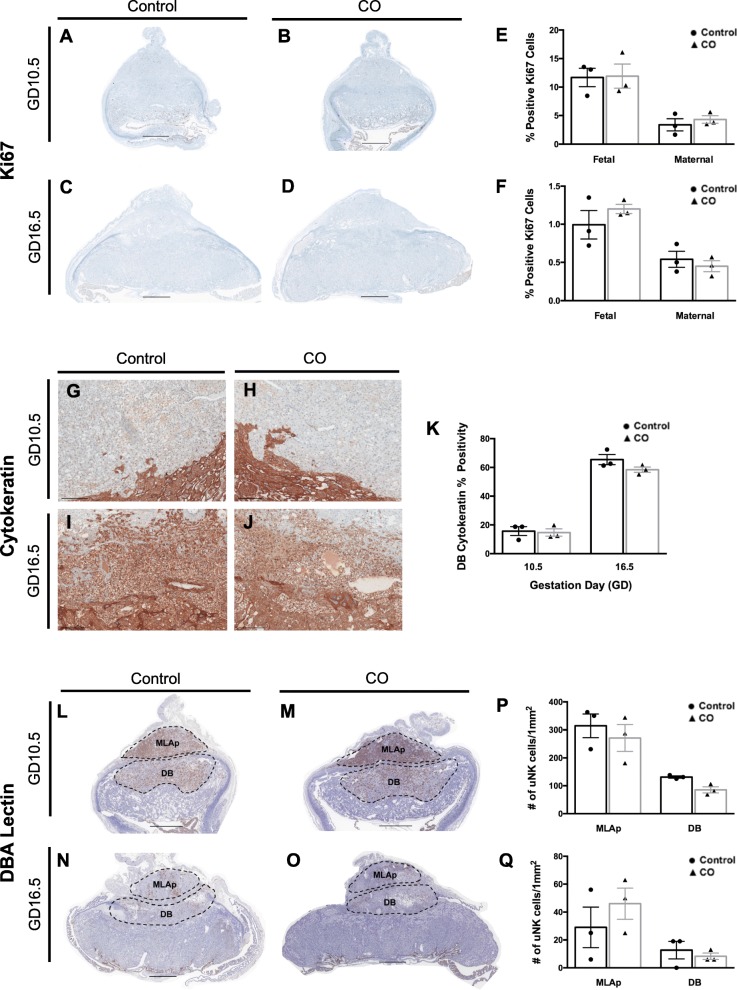
Effect of CO on cellular proliferation, trophoblast invasion, and uNK cell abundance. (**a**-**d**) Representative images of whole implantation sites at GD10.5 and GD16.5 immunostained with Ki67, a cell proliferation marker (2X; scale bar, 1000 μm). Semi-quantitative analysis of % positive Ki67 cells in fetal and maternal areas of implantation sites on (**e**) GD10.5 or (**f**) GD16.5, from dams treated with CO compared to controls. Fetal area was defined as the Lab and JZ, while maternal area was defined as the DB and MLAp. (**g**-**j**) Representative images of trophoblast cell invasion into the DB on GD10.5 and GD16.5 (10X; scale bar, 200 μm). (**k**) Semi-quantitative analysis of pan-cytokeratin staining in the DB on GD10.5 and GD16.5 in mice exposed to CO. (**l**-**o**) Representative images of whole implantation sites at GD10.5 and GD16.5 immunostained with DBA lectin, a marker of uNK cells (2X; scale bar, 1000 μm). Semi-quantitative analysis of uNK cells in the MLAp or DB at (**p**) GD10.5 or (**q**) GD16.5. Data are expressed as mean ± SEM. Statistical analysis was performed using the Mann-Whitney U test. A *p* value< 0.05 was used to determine statistical significance. (*n* = 3 control, *n* = 3 CO at GD10.5 and GD16.5). CO, carbon monoxide; DB, decidua basalis; GD, gestation day; JZ, junctional zone; Lab, labyrinth; MLAp, mesometrial lymphoid aggregate of pregnancy; uNK, uterine natural killer

To determine the impact of CO on pregnancy specific adaptations, immunohistochemistry was performed on GD10.5 and GD16.5 implantation sites to determine the effect of CO on cellular proliferation (Ki67), trophoblast invasion (pan-cytokeratin), and uNK cell infiltration (DBA lectin cytochemistry). To assess whether CO would impact the normal cellular turnover in the placenta, we investigated Ki67 staining in the maternal and fetal regions of the implantation site. When analyzing the percent positive Ki67 cells in the MLAp and DB, and the JZ and Lab zones, we detected no measurable differences in implantation sites between CO treated and control mice (Fig. 2a-f), suggesting that CO does not alter this parameter of placental health. Additionally, no changes were detected in cytokeratin percent positivity in the DB, indicating that CO does not appear to alter normal trophoblast invasion (Fig. 2g-k). Uterine NK cells have important immunological and angiogenic roles at the maternal-fetal interface [21]. To demonstrate the effect of CO on DBA^+^ uNK cell numbers during and following their midgestation peak, DBA lectin staining was performed at GD10.5 and GD16.5, respectively. We found that the uNK cell abundance was similar in the MLAp and DB of CO treated and control mice both mid- and late-gestation (Fig. 2l-q), suggesting that in a healthy pregnancy, CO does not modify the uNK cell population. Collectively, these results suggest that CO does not impact these measures of placental health during murine pregnancy.

## Conclusions

In summary, the present study supports the notion that CO potentiates utero-placental vascular growth through major angiogenic pathways without impacting pregnancy specific adaptations. These results reinforce the continued research into CO administration, or manipulation of endogenous CO production, to increase angiogenesis at the maternal-fetal interface for pregnancies at risk of or complicated by PE and IUGR.

## Supplementary information

**Additional file 1: Table S1.** Custom RT^2^ Profiler Array containing 84 angiogenic and inflammatory related genes used to quantify gene expression at the implantation site on GD10.5 and GD16.5.

**Additional file 2: Table S2.** Pregnancy outcomes in carbon monoxide and control treated dams on GD10.5 and GD16.5. Number of implantation sites, live fetuses, and fetal resportions per litter in CO treated mice at GD10.5 (*n* = 5 control, *n* = 5 CO) and GD16.5 (*n* = 5 control, *n* = 4 CO). Data are presented as mean ± SEM, analyzed by the Mann-Whitney U test. A *p* value< 0.05 was used to determine statistical significance; no significance was found between any of the pregnancy outcomes measured. CO, carbon monoxide; GD, gestation day

**Additional file 3: Figure S1.** Effect of carbon monoxide on maternal plasma cytokine profile throughout gestation. Maternal plasma cytokine levels of CO exposed and control mice on GD0.5 (*n* = 10 control, *n* = 9 CO), GD5.5 (*n* = 10 control, *n* = 9 CO), GD10.5 (*n* = 10 control, *n* = 9 CO), and GD16.5 (*n* = 5 control, *n* = 4 CO). Data are expressed as mean ± SEM, and analyzed by the Mann-Whitney U test; treatment groups were compared at each time point and a Bonferroni correction was used to compare the family wise error rate. An overall *p* value of 0.05 was used, with a p value cut off of 0.0125 for each of the four individual pairwise comparisons. CO, carbon monoxide; GD, gestation day

**Additional file 4: Figure S2.** Cytokine profile of GD10.5 implantation sites of control and carbon monoxide treated mice. Cytokine concentrations at the implantation sites of CO exposed and control mice on GD10.5 (*n* = 5 control, *n* = 5 CO). Data are presented as mean ± SEM, analyzed by the Mann-Whitney U test. A *p* value< 0.05 was used to determine statistical significance; no significance was found between treatment groups. CO, carbon monoxide; GD, gestation day

## Data Availability

All data generated or analyzed during this study are included in this published article.

## Abbreviations

CO: Carbon monoxide
COHb: Carboxyhemoglobin
DB: Decidua basalis
DBA: *Dolichos biflorus* agglutinin
GD: Gestation day
IUGR: Intrauterine growth restriction
JZ: Junctional zone
Lab: Labyrinth
MLAp: Mesometrial lymphoid aggregate of pregnancy
PE: Preeclampsia
uNK: Uterine natural killer
