# Supplementary material for: Carbon monoxide increases utero-placental angiogenesis without impacting pregnancy specific adaptations in mice
Source: Reprod Biol Endocrinol. 2020 May 14;18:49. doi: 10.1186/s12958-020-00594-z (PMC7227344; doi:10.1186/s12958-020-00594-z)
Supplement: Supplementary file 1 — Additional file 1: Table S1. Custom RT2 Profiler Array containing 84 angiogenic and inflammatory related genes used to quantify gene expression at the implantation site on GD10.5 and GD16.5. [file 12958_2020_594_MOESM1_ESM.pdf]

Vegfa	*Vegfb*	*Vegfc*	*Figf*	*Eng*	*Flt1*	*Kdr*
Flt4	*Pgf*	*Angpt1*	*Angpt2*	*Fgf1*	*Fgf2*	*Pdgfa*
Egf	*Tgfa*	*Tgfb1*	*Tgfb2*	*Tgfb3*	*Hgf*	*Igf1*
Tymp	*Ccl2*	*Cxcl1*	*Ang*	*Plg*	*Timp1*	*Timp2*
Nos3	*Cdh5*	*Ephb4*	*Efnb2*	*Efna1*	*Tie1*	*Tek*
Dll4	*Notch1*	*Plau*	*Tnf*	*Csf1*	*Csf2*	*Csf3*
Pecam1	*Sox11*	*Prl4a1*	*Bag1*	*Bcl2*	*Bcl2l1*	*Akt1*
Nfe2l2	*Nrp1*	*Thbs1*	*Thbs2*	*Col18a1*	*Bai1*	*Pf4*
Hif1a	*Hif1an*	*Arnt*	*Hmox1*	*Hmox2*	*Cxcl10*	*Cxcl12*
Ccl11	*Cx3cl1*	*Ifng*	*Il1a*	*Il1b*	*Il6*	*Il10*
Il15	*Il16*	*Il17a*	*Il18*	*Lif*	*Ccl4*	*Ccl3*
Epo	*Nos2*	*Plxdc1*	*Nfkb1*	*Ccl12*	*Pdgfb*	*Dcn*

Additional Table 1. Custom RT^2 Profiler Array containing 84 angiogenic and inflammatory related genes used to quantify gene expression at the implantation site on GD10.5 and GD16.5.
