# Supplementary material for: Carbon monoxide increases utero-placental angiogenesis without impacting pregnancy specific adaptations in mice
Source: Reprod Biol Endocrinol. 2020 May 14;18:49. doi: 10.1186/s12958-020-00594-z (PMC7227344; doi:10.1186/s12958-020-00594-z)
Supplement: Supplementary file 2 — Additional file 2: Table S2. Pregnancy outcomes in carbon monoxide and control treated dams on GD10.5 and GD16.5. Number of implantation sites, live fetuses, and fetal resportions per litter in CO treated mice at GD10.5 (n = 5 control, n = 5 CO) and GD16.5 (n = 5 control, n = 4 CO). Data are presented as mean ± SEM, analyzed by the Mann-Whitney U test. A p value< 0.05 was used to determine statistical significance; no significance was found between any of the pregnancy outcomes measured. CO, carbon monoxide; GD, gestation day [file 12958_2020_594_MOESM2_ESM.pdf]

Additional Table 2: Pregnancy outcomes in carbon monoxide and control treated dams on GD10.5 and GD16.5.

	**Mean number of implantation sites per litter (±SEM)**		**Mean number of fetuses per litter (±SEM)**		**Mean number of resportions per litter (±SEM)**	
	Control	CO	Control	CO	Control	CO
GD10.5	13.2 (0.70)	14.4 (0.24)	12.6 (0.87)	14.4 (0.24)	0.5 (0.22)	0 (0)
GD16.5	13.2 (0.58)	14.8 (1.25)	11.8 (0.73)	12.8 (1.65)	1.4 (0.68)	2.0 (0.91)

Number of implantation sites, live fetuses, and fetal resportions per litter in CO treated mice at GD10.5 (n=5 control, n=5 CO) and GD16.5 (n=5 control, n=4 CO). Data are presented as mean±SEM, analyzed by the Mann-Whitney U test. A p value<0.05 was used to determine statistical significance; no significance was found between any of the pregnancy outcomes measured. CO, carbon monoxide; GD, gestation day
