# Supplementary material for: Carbon monoxide increases utero-placental angiogenesis without impacting pregnancy specific adaptations in mice
Source: Reprod Biol Endocrinol. 2020 May 14;18:49. doi: 10.1186/s12958-020-00594-z (PMC7227344; doi:10.1186/s12958-020-00594-z)
Supplement: Supplementary file 3 — Additional file 3: Figure S1. Effect of carbon monoxide on maternal plasma cytokine profile throughout gestation. Maternal plasma cytokine levels of CO exposed and control mice on GD0.5 (n = 10 control, n = 9 CO), GD5.5 (n = 10 control, n = 9 CO), GD10.5 (n = 10 control, n = 9 CO), and GD16.5 (n = 5 control, n = 4 CO). Data are expressed as mean ± SEM, and analyzed by the Mann-Whitney U test; treatment groups were compared at each time point and a Bonferroni correction was used to compare the family wise error rate. An overall p value of 0.05 was used, with a p value cut off of 0.0125 for each of the four individual pairwise comparisons. CO, carbon monoxide; GD, gestation day [file 12958_2020_594_MOESM3_ESM.pdf]

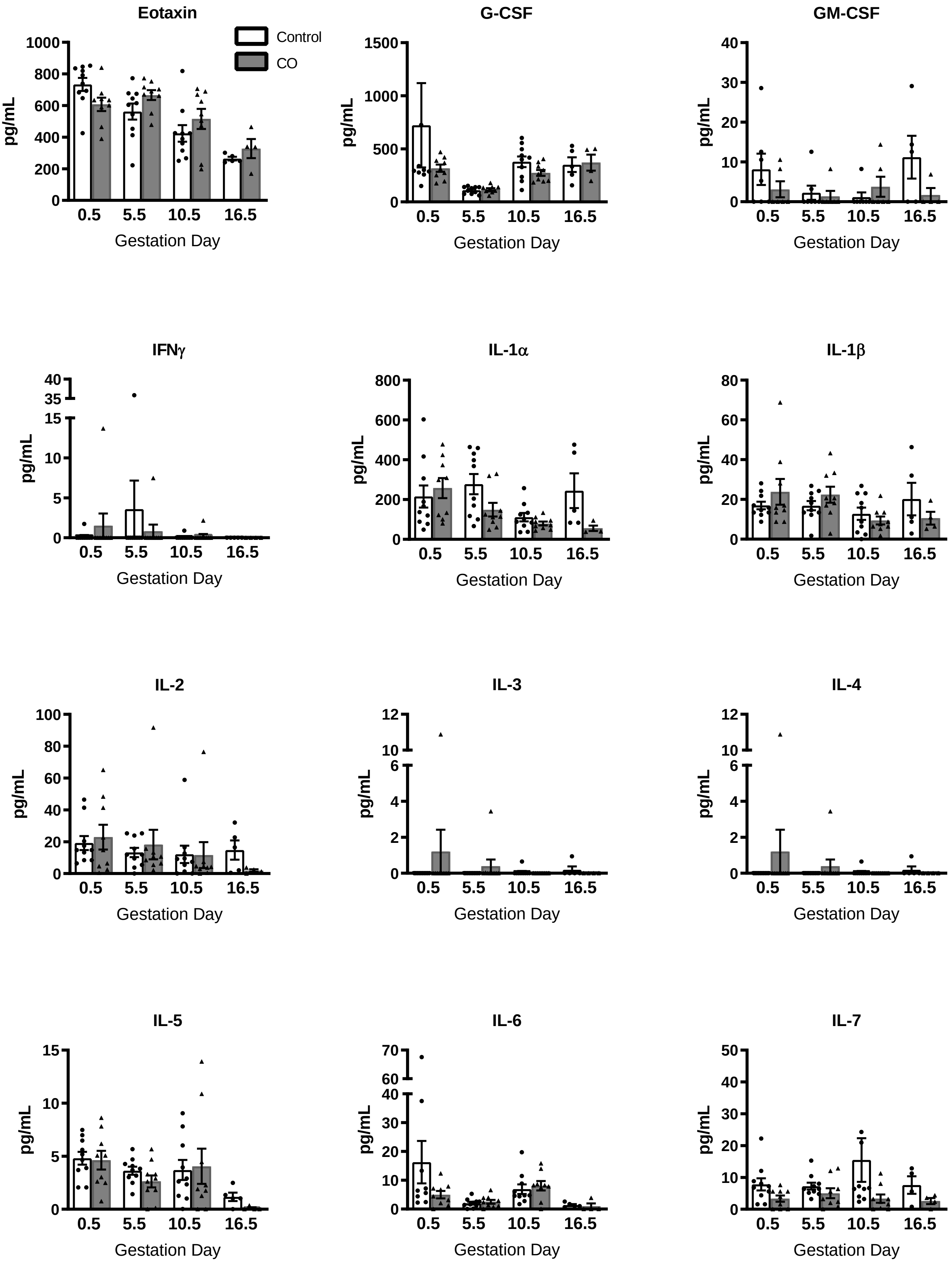
Eotaxin
Control
CO
G-CSF
GM-CSF
IFNγ
IL-1α
IL-1β
IL-2
IL-3
IL-4
IL-5
IL-6
IL-7
pg/mL
0.5
5.5
10.5
16.5
Gestation Day

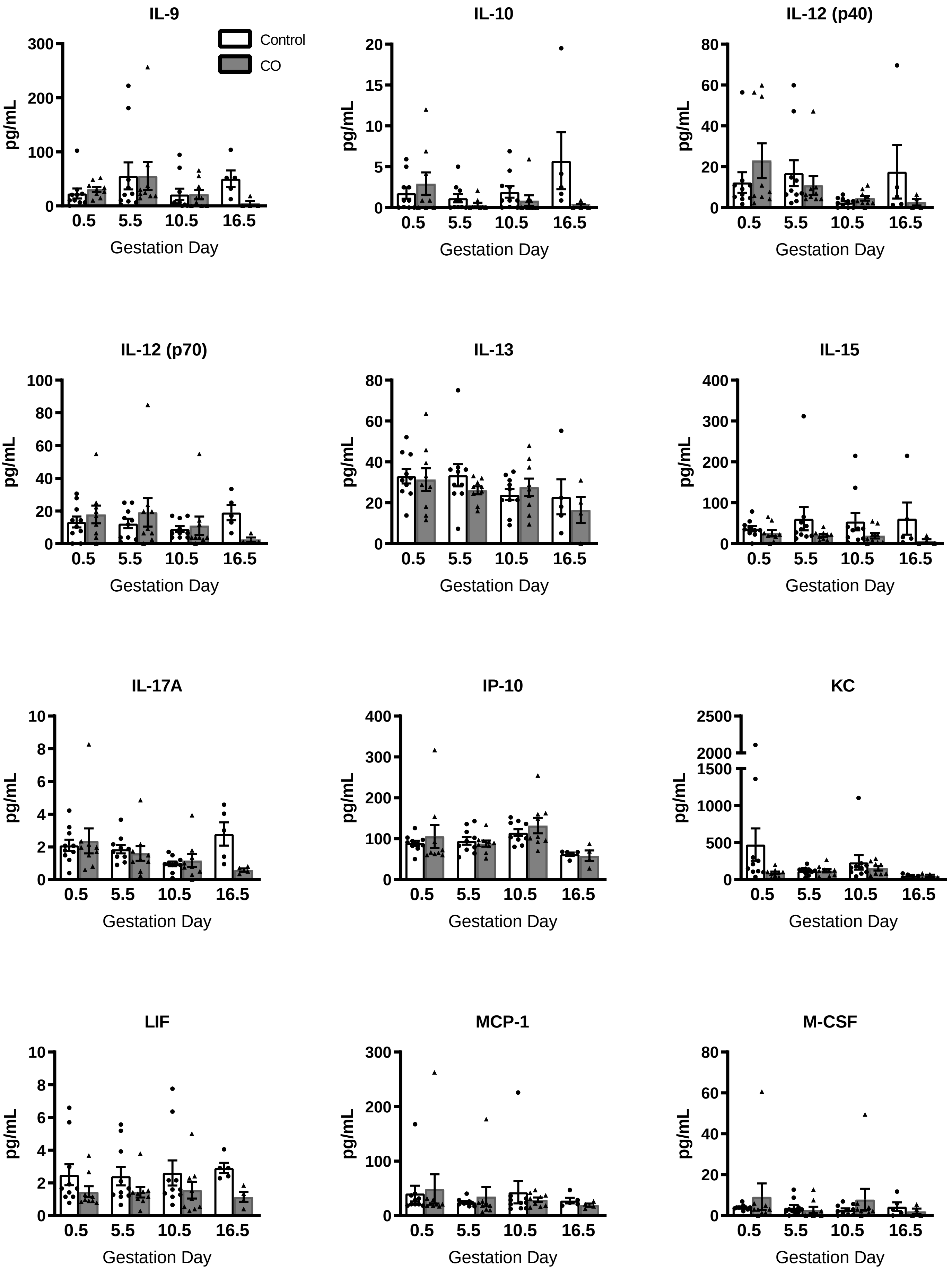
IL-9
Control
CO
IL-10
IL-12 (p40)
IL-12 (p70)
IL-13
IL-15
IL-17A
IP-10
KC
LIF
MCP-1
M-CSF
pg/mL
Gestation Day
0.5
5.5
10.5
16.5

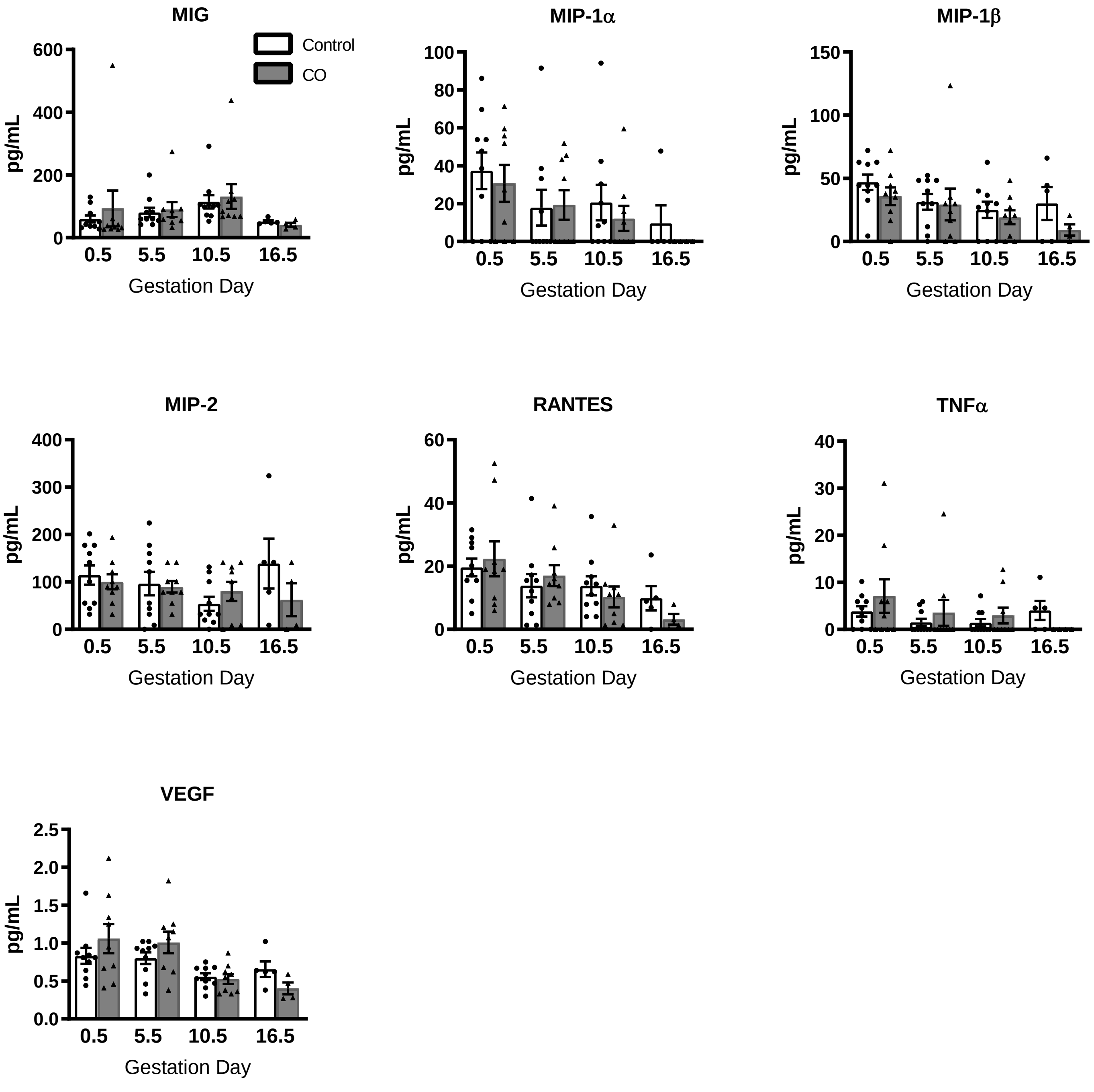

Additional Figure 1. Effect of carbon monoxide on maternal plasma cytokine profile throughout gestation. Maternal plasma cytokine levels of CO exposed and control mice on GD0.5 (n=10 control, n=9 CO), GD5.5 (n=10 control, n=9 CO), GD10.5 (n=10 control, n=9 CO), and GD16.5 (n=5 control, n=4 CO). Data are expressed as mean±SEM, and analyzed by the Mann-Whitney U test; treatment groups were compared at each time point and a Bonferroni correction was used to compare the family wise error rate. An overall p value of 0.05 was used, with a p value cut off of 0.0125 for each of the four individual pairwise comparisons. CO, carbon monoxide; GD, gestation day
