# Supplementary material for: Carbon monoxide increases utero-placental angiogenesis without impacting pregnancy specific adaptations in mice
Source: Reprod Biol Endocrinol. 2020 May 14;18:49. doi: 10.1186/s12958-020-00594-z (PMC7227344; doi:10.1186/s12958-020-00594-z)
Supplement: Supplementary file 4 — Additional file 4: Figure S2. Cytokine profile of GD10.5 implantation sites of control and carbon monoxide treated mice. Cytokine concentrations at the implantation sites of CO exposed and control mice on GD10.5 (n = 5 control, n = 5 CO). Data are presented as mean ± SEM, analyzed by the Mann-Whitney U test. A p value< 0.05 was used to determine statistical significance; no significance was found between treatment groups. CO, carbon monoxide; GD, gestation day [file 12958_2020_594_MOESM4_ESM.pdf]

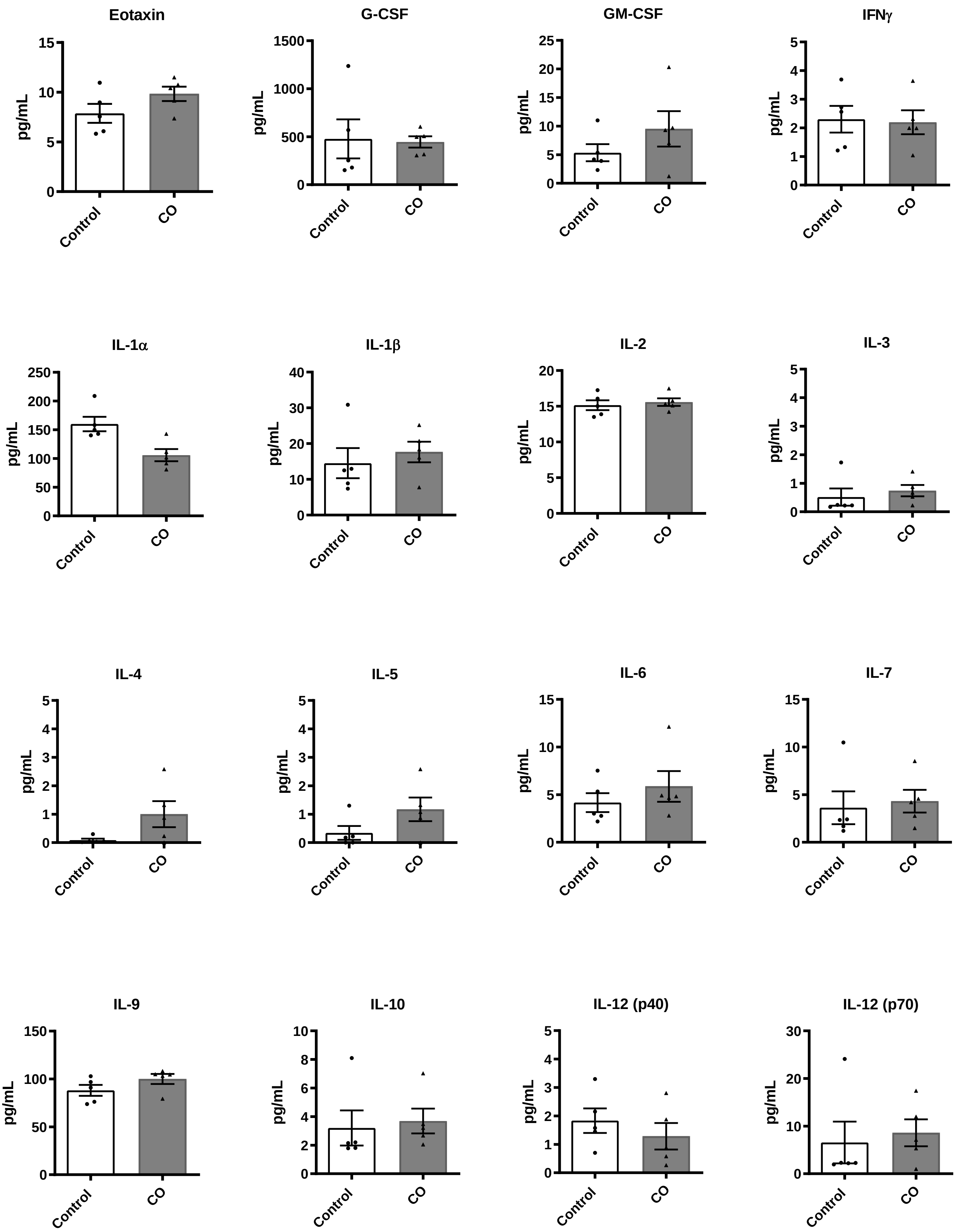

Eotaxin
15
10
5
0
pg/mL
Control
CO
G-CSF
1500
1000
500
0
pg/mL
Control
CO
GM-CSF
25
20
15
10
5
0
pg/mL
Control
CO
IFNγ
5
4
3
2
1
0
pg/mL
Control
CO
IL-1α
250
200
150
100
50
0
pg/mL
Control
CO
IL-1β
40
30
20
10
0
pg/mL
Control
CO
IL-2
20
15
10
5
0
pg/mL
Control
CO
IL-3
5
4
3
2
1
0
pg/mL
Control
CO
IL-4
5
4
3
2
1
0
pg/mL
Control
CO
IL-5
5
4
3
2
1
0
pg/mL
Control
CO
IL-6
15
10
5
0
pg/mL
Control
CO
IL-7
15
10
5
0
pg/mL
Control
CO
IL-9
150
100
50
0
pg/mL
Control
CO
IL-10
10
8
6
4
2
0
pg/mL
Control
CO
IL-12 (p40)
5
4
3
2
1
0
pg/mL
Control
CO
IL-12 (p70)
30
20
10
0
pg/mL
Control
CO

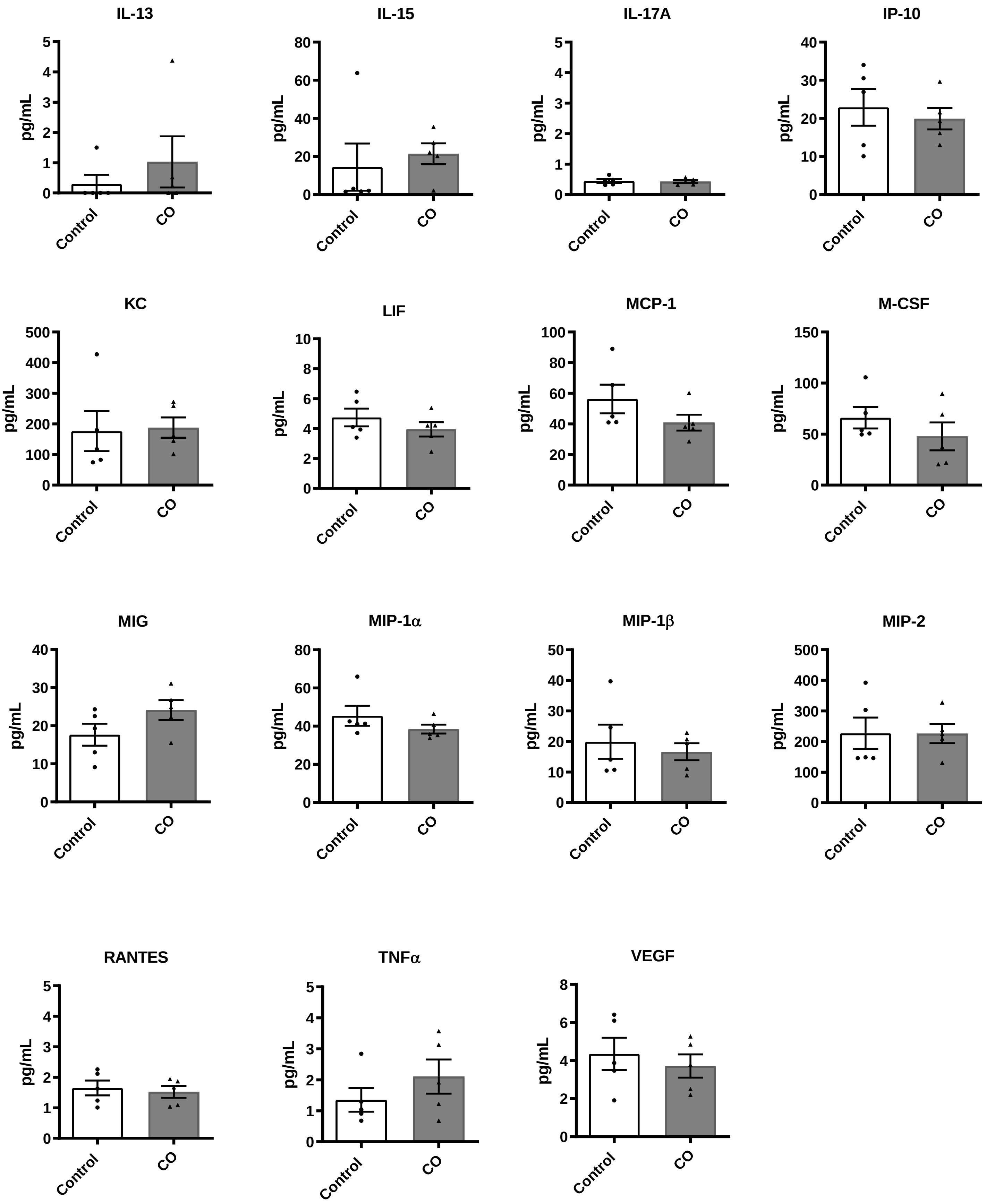

Additional Figure 2. Cytokine profile of GD10.5 implantation sites of control and carbon monoxide treated mice. Cytokine concentrations at the implantation sites of CO exposed and control mice on GD10.5 (n=5 control, n=5 CO). Data are presented as mean±SEM, analyzed by the Mann-Whitney U test. A p value<0.05 was used to determine statistical significance; no significance was found between treatment groups. CO, carbon monoxide; GD, gestation day.
